# Particle length-dependent titanium dioxide nanomaterials toxicity and bioactivity

**DOI:** 10.1186/1743-8977-6-35

**Published:** 2009-12-31

**Authors:** Raymond F Hamilton, Nianqiang Wu, Dale Porter, Mary Buford, Michael Wolfarth, Andrij Holian

**Affiliations:** 1 Center for Environmental Health Sciences, University of Montana, Missoula MT, USA; 2 Mechanical and Aerospace Engineering, WV Nano Initiative, West Virginia University, Morgantown, WV 26506-6106, USA; 3 Health Effects Laboratory Division, NIOSH, Morgantown, VW, USA

## Abstract

**Background:**

Titanium dioxide (TiO_2_) nanomaterials have considerable beneficial uses as photocatalysts and solar cells. It has been established for many years that pigment-grade TiO_2 _(200 nm sphere) is relatively inert when internalized into a biological model system (in vivo or in vitro). For this reason, TiO_2 _nanomaterials are considered an attractive alternative in applications where biological exposures will occur. Unfortunately, metal oxides on the nanoscale (one dimension < 100 nm) may or may not exhibit the same toxic potential as the original material. A further complicating issue is the effect of modifying or engineering of the nanomaterial to be structurally and geometrically different from the original material.

**Results:**

TiO_2 _nanospheres, short (< 5 μm) and long (> 15 μm) nanobelts were synthesized, characterized and tested for biological activity using primary murine alveolar macrophages and in vivo in mice. This study demonstrates that alteration of anatase TiO_2 _nanomaterial into a fibre structure of greater than 15 μm creates a highly toxic particle and initiates an inflammatory response by alveolar macrophages. These fibre-shaped nanomaterials induced inflammasome activation and release of inflammatory cytokines through a cathepsin B-mediated mechanism. Consequently, long TiO_2 _nanobelts interact with lung macrophages in a manner very similar to asbestos or silica.

**Conclusions:**

These observations suggest that any modification of a nanomaterial, resulting in a wire, fibre, belt or tube, be tested for pathogenic potential. As this study demonstrates, toxicity and pathogenic potential change dramatically as the shape of the material is altered into one that a phagocytic cell has difficulty processing, resulting in lysosomal disruption.

## Background

There is an abundance of potential uses for TiO_2_, which increase as the TiO_2 _is converted to a nanomaterial [1]. Pigment grade titanium dioxide is widely used as a pigment due to its brightness and high refractive index. It can be found in paints, plastics, paper, inks, foods, medicines (pills), and toothpaste. A very common application of TiO_2 _is as an additive in sunscreen cosmetics because it acts as a sink for UV exposure, converting the UV light to heat [2]. Other uses include being a functional part in some oxygen sensors, bone/medical implant integration, cleaving proteins at proline sites [3], odor controller in cat litter, and as a semiconductor [2]. In recent years, with the development of nanotechnology, TiO_2 _nanobelts are finding increasing applications as photocatalysts [4], and TiO_2 _nanowires have uses in solar cells [5].

For many years TiO_2 _has been considered to be biologically inert, suggesting that environmental or occupational exposure of the material, regardless of exposure route, was relatively harmless and easily and effectively processed out of the body. With the advent of nanotechnology some of these assumptions of safety would be challenged [6,7]. In particular, the TiO_2 _material could be engineered in terms of shapes and sizes. The reduction of the particle size leads to higher specific surface area. Tailoring sphere-shaped nanoparticles to fibre-shaped nanoparticles such as nanowires, nanobelts and nanotubes is very attractive [8,9], because fibre-shaped nanomaterials have advantages in the application of photocatalysis, charge transfer and sensing due to its unique structure. Preliminary toxicological studies have produced conflicting results with regard to the toxic potential of these engineered materials depending on the biological model and material used.

In vivo studies showed that rats instilled with anatase nanorods and nanodots did not produce lung inflammation or pathological changes differing from pigment-grade TiO_2 _indicating that the increased surface area of the nano-sized TiO_2 _had no effect on toxicity [10]. This observation was confirmed using nanoquartz and quartz in a similar study [11]. In another study, the same group attributed observed differences in the toxicity of ultrafine TiO_2 _particles to differences in rutile/anatase surface properties [12]. Another in vivo study exposing mice to TiO_2 _nanoparticles (2 to 5 nm) was essentially negative showing a reversible inflammation characterized by an increase in alveolar macrophages (AM) in lungs [13]. A recent study using mice injected repeatedly with TiO_2 _(5 nm) nanoparticles in the abdominal cavity suggested that inflammatory damage was limited to the organs where the TiO_2 _nanoparticles accumulated over time, namely the liver, kidney and myocardium of the exposed mice [14]. A similar study in mice using variable TiO_2 _dosages came to the same conclusion with the exception of the spleen and lung being added to the list of organs where the nanoparticles accumulate [15].

In contrast, another study using fibrous TiO_2 _compared to pigment-grade TiO_2 _exposed to rat macrophages showed that the fibrous form of the TiO_2 _was much more cytotoxic, leading this group to conclude that TiO_2 _toxicity was dependent on the shape of the particle being processed by the macrophage [16]. Other claims of damage seen in TiO_2 _ultrafine particle exposures in vitro include hydrogen peroxide release and oxidative DNA damage in a human bronchial epithelial cell line [17], and TiO_2 _nanoparticles generated genotoxicity and cytotoxicty in a cultured human cell line (WIL2-NS) [18]. The only study modeling exposure risk in humans (manufacturing workers) exposed to TiO_2 _nanoparticles concluded there would be physiological effects of TiO_2 _inhalation (increased neutrophils in the lung), but that it would not pose a significant cancer risk [19]. Therefore, based on the immporance of these nanomaterials and the suggestion that long materials could be more toxic we tested the hypothesis that length may be an important determinant of nanomaterial biocompatibility,

## Results

### Characterization of Anatase Titanium Dioxide Nanomaterials

The particles in this study were synthesized as described in Methods and characterized as follows. The particle morphology was observed with a Hitachi S4700 field-emission scanning electron microscopy (SEM). The crystal structure of the TiO_2 _particles was characterized by X-ray diffraction with Cu *Kα *radiation (XRD, X'Pert Pro PW3040-Pro, Panalytical Inc.) and high resolution transmission electron microscopy (HRTEM), a 200 kV FEI/Philips CM20 apparatus). For TEM sample preparation, the TiO_2 _powders were suspended in ethanol. The suspension was then dropped onto a holey carbon film supported by a copper grid, subsequently dried in air prior to TEM observation.

**Figure 1:**
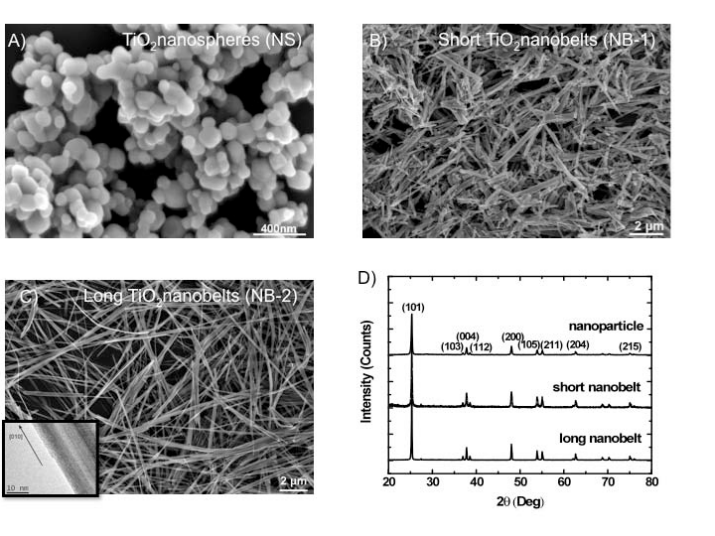
**SEM and XRD analyses confirm morphology and crystal structure of three TiO_2 _nanoparticles differing in geometric dimension**. **A**, Image of titanium dioxide nanospheres (NS) 60-200 nm in diameter. **B**, Image of titanium dioxide short nanobelts (NB-1) 60-300 nm in diameter and 0.8-4 μm in length. **C**, Image of titanium dioxide long nanobelts (NB-2) 60-300 nm in diameter and 15-30 μm in length. **D**, XRD patterns of all three titanium dioxide nanoparticles stacked. The data was consistent with all three nanoparticles exhibiting anatase structure.

Figure 1(a - c) shows the SEM images taken from the three types of TiO_2 _nanoparticles. The nanospheres (NS) are in a diameter of 60 ~ 200 nm. The width of both the long and the short nanobelts are in the range of 60 ~ 300 nm. Most of the long nanobelts (NB-2) are about 15 ~ 30 μm long and the short nanobelts (NB-1) are about 0.8 ~ 4 μm long. All three types of TiO_2 _nanoparticles exhibit a monolithic anatase phase as demonstrated by the XRD patterns in Figure 1d. The HRTEM analysis confirmed that the nanobelts are single crystalline anatase TiO_2 _with the growth direction along [010]. Figure 1c (inset) shows the lattice fringes perpendicular to the growth direction with a space of 0.38 nm, which represents the lattice parameter of 0.38 nm in the [010] direction. Zeta potentials of the TiO_2 _nanoparticles in the media used for in vitro experiments were as follows: NS (-11.7 mV), NB-1 (-12.06 mV), and NB-2 (-11.33 mV).

### Characterization of Cell/Particle interaction

**Figure 2:**
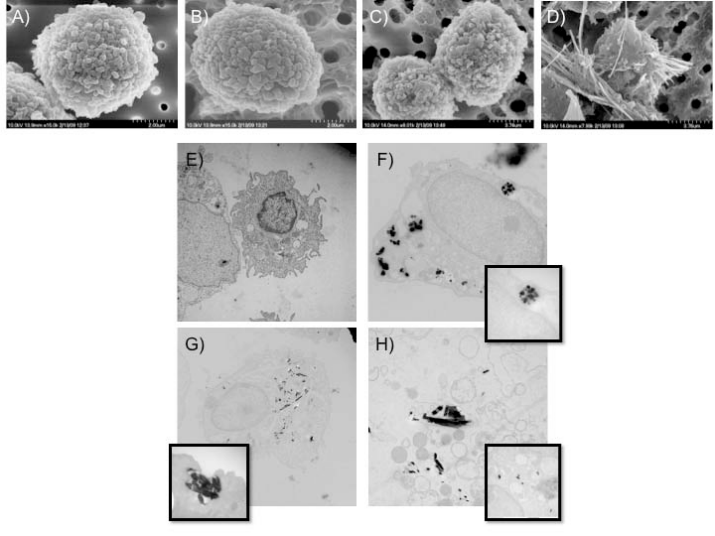
***In vitro *cell/particle interaction captured by SEM (to image the outside of cell), and TEM (to image the inside of the cell) following a 1 hour particle exposure**. **A**, SEM and (**E**) TEM of unexposed control C57BL/6 alveolar macrophage (AM). **B**, The SEM and (**F**) TEM of TiO_2 _NS-exposed AM revealed a high concentration of particle collection in the cytoplasm of the AM, compartmentalized in lysosomal structures (**inset**). The SEM revealed no external NS concentration. **C**, The SEM and (**G**) TEM of NB-1-exposed AM revealed a similar high concentration of particle collection in the cytoplasm of the AM again compartmentalized in lysosomal structures (**inset**). The SEM revealed very few NB-1 on the cell surface. **D**, The SEM of the TiO_2 _long nanobelt-exposed AM (NB-2) showed many belts on the outside of the cell, as the (**H**) TEM image illustrated that these belts are also internalized to some degree. The TEM images also suggest there are fewer lysosomal structures associated with the NB-2 exposure in addition to an increased number of belts segments directly exposed to the cell's cytoplasm (**inset**).

Examination of cell/particle interaction using SEM and TEM provided the first clue of how the long nanobelts are processed or maybe better described as misprocessed. Figure 2(a - d) shows the outside of an AM after being exposed to TiO_2 _nanoparticle for 1 hour in a suspension culture. The AM exposed to NS and NB-1 appeared normal with no evidence of particles on the outside of the cell. In contrast, the AM exposed to NB-2 showed many belts external to the body of the cell with some nanobelts superficially attached to the cell surface, and several belts going through the body of the cell.

The TEM images in Figure 2(e - h), show that the NS were taken up in the cytoplasm in discrete lysosomes (Figure 2f inset). Similarly, the NB-1 were also taken up in to discrete lysosomes that were formed by the plasma membrane engulfing several nanobelts and then sequestering the material into a future lysosome (Figure 2g inset). In contrast, the AM exposed to NB-2 failed to produce functional lysosomal domains. The long belts were internalized to a degree, but are visible "free-floating" in the cytoplasm of the cell (Figure 2h inset). We propose that the AM attempts to form discrete lysosomes around these long belts, but because of the length of the belt, the lysosomes become unstable and as a result destructive enzymes such as cathepsin B are released into the cytoplasm and eventually into the media.

### Titanium Dioxide NB uptake is Not Mediated By MARCO Receptor

**Figure 3:**
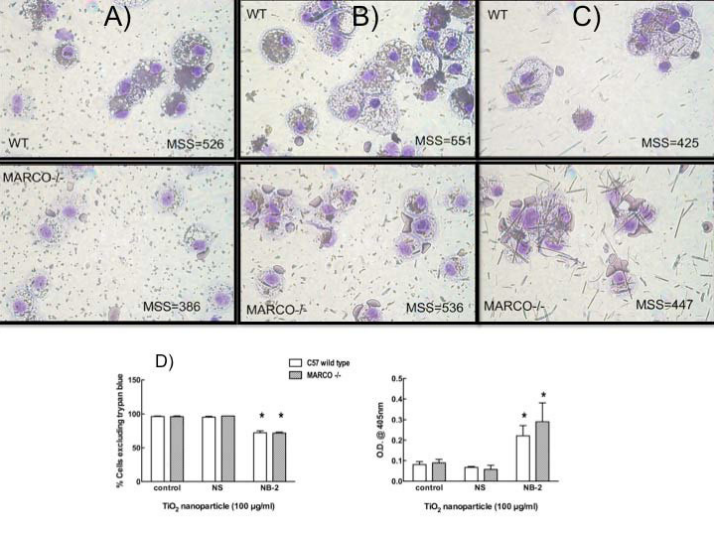
**The macrophage MARCO receptor is uniquely employed to bind and take up the TiO_2 _nanospheres (NS), but has no recognition for the short (NB-1) or long (NB-2) TiO_2 _nanobelts**. **A**, Bright-field microscopy showing uptake of NS in C57BL/6 wildtype AM. Binding and uptake of the NS was significantly hindered in the AM from MARCO null mice. In contrast, **B **and **C **panels illustrate no binding or uptake problems in MARCO-deficient AM exposed to either short (NB-1) or long (NB-2) TiO_2 _nanobelts. MSS values indicate 'median side scatter' which was used as a metric for particle binding. Panel **D **illustrates the point further, as it shows that the loss of viability and increased apoptosis associated with NB-2 exposure is not affected by MARCO expression on AM. Data expressed as mean ± *SEM*. Asterisk (*) indicates *P *< 0.05 compared to control.

Experiments using AM from MARCO null mice indicate that the receptor is involved in the binding and uptake of the NS only, but not the NB-1 or NB-2. Figure 3a demonstrates the difference in NS uptake between C57BL/6 wild-type AM and AM's from MARCO null mice. In contrast, there is no obvious difference between the amount of nanoparticle taken up by the wild-type or MARCO null AM as determined by side scatter associated with the cell/particle being processed by flow cytometry (Figures 3b and 3c). Additionally, the toxicity of NB-2 was not diminished in the absence of the MARCO receptor further indicating that this receptor was not involved in the uptake of the longer TiO_2 _material (Figure 3d).

### Alveolar Macrophage Toxicity by the Long Titanium Dioxide NB-2

**Figure 4:**
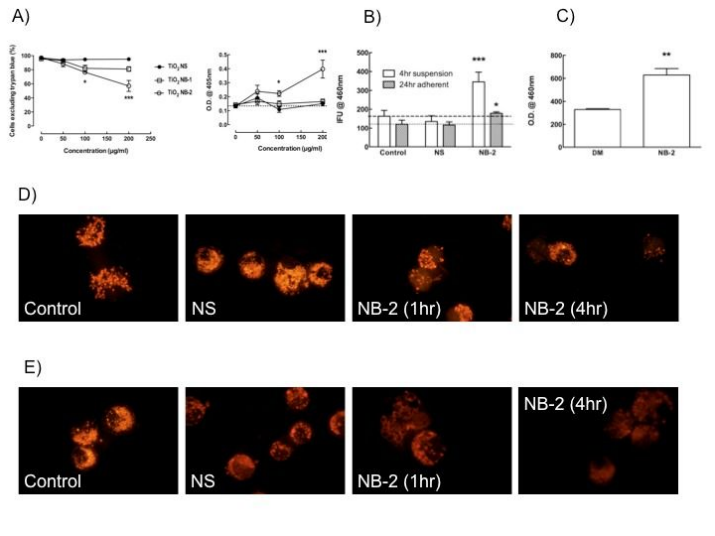
**The mechanistic basis for TiO_2 _long nanobelt (NB-2)-induced toxicity to alveolar macrophages (AM) is lysosomal breakdown resulting in cathepsin B release**. **A**, panels illustrate the concentration-dependent cell death uniquely initiated by NB-2 exposure in the C57BL/6 AM. Cell viability by trypan blue exclusion is on the right, and measured apoptosis is on the left. **B**, corresponding cathepsin activity in the AM media showed significant increases in cultures that were exposed to NB-2. **C**, This is supported by the *in vivo *observation that cathepsins are significantly increased in the lavage fluid of mice 24 hours following NB-2 instillation (30 μg/mouse). Panels in **D**, show acridine orange-stained lysosomes in unstimulated cells, NS-exposed AM, early (1hr) NB-2-exposed AM and late (4 hr) NB-2-exposed AM, respectively. The NS-exposure appears to concentrate the lysosomes in the AM, whereas the NB-2-exposure initially causes reorganization of the lysosomes and subsequently depletes the lysosomes from the AM. (**E**), similar patterns were visualized using a fluorescent cathepsin B substrate under identical assay conditions as in (**D**). The NS-exposure appears to concentrate the cathepsin B in the AM, whereas the NB-2-exposure depletes the cathepsin in the exposed AM. Data expressed as mean ± *SEM*. Asterisk (*) indicates *P *< 0.05, double asterisks (**) indicates *P *< 0.01, and triple asterisks (***) indicates *P *< 0.001 compared to baseline or control production levels.

The relative cytotoxicity of the three forms of TiO_2 _can be found in Figure 4a with measurement of cell viability and apoptosis in a 4-hour suspension culture. Only the NB-2 exposure was significantly cytotoxic at the 100 and 200 μg/ml concentrations. NS and NB-1 were not significantly cytotoxic. The mechanism of cytotoxicity briefly discussed earlier involves the loss of lysosomal integrity and the subsequent release of cathepsin B. Figure 4b illustrates the cathepsin activity in the media following a 4-hour suspension culture or a 24-hour adherent culture. Regardless of the way the AM are cultured the NB-2 exposure causes a significant release of cathepsin into the media compared to baseline release. Figure 4c shows a significant increase of cathepsins in the lavage fluid of mice exposed to 30 μg of NB-2 for 24 hours compared to DM vehicle.

Fluorescent imaging of the lysosomes in AM exposed to TiO_2 _nanoparticles using acridine orange illustrates the process (Figure 4d). Internalization of the NS causes a concentration of the lysosomes to appear in the AM. In contrast, the NB-2 exposure causes a brief reorganization (1 hr) followed by a degredation/depletion of the lysosomes in some, but not all AM (4 hr). A similar process occurs with the cathepsin B imaged by a fluorescent substrate (Figure 4e). The cathepsin B substrate becomes visibly diffuse in AM exposed to NB-2 regardless of the culture timing, whereas cathepsin B substrate in AM exposed to NS is more concentrated and isolated in the lysosomes.

### All forms of Titanium Dioxide Nanomaterials Caused Reactive Oxygen Species in the Alveolar Macrophage

**Figure 5:**
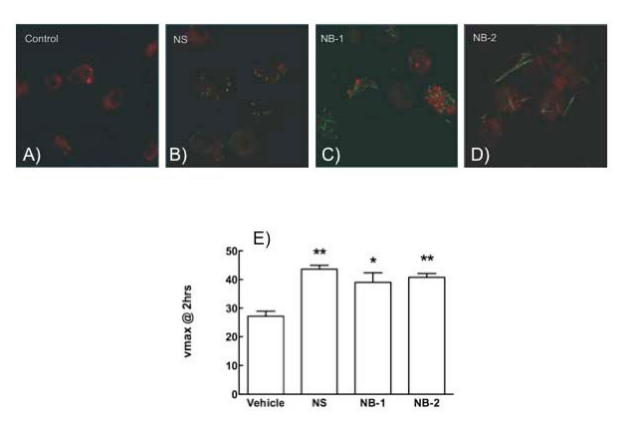
**All forms of TiO_2 _cause reactive oxygen species (ROS) generation**. The relative lipid peroxidation of the three TiO_2 _nanoparticles using a fluorescent BODIPY stain in alveolar macrophages, which changes to green from red in the presence of oxygen radical damage. **A**: no particle control, **B**: NS, **C**: NB-1, and **D**: NB-2. All three nanoparticles produce some degree of lipid peroxidation indicated by the presence of green stain. In addition, intracellular ROS were measured over a 2 hour nanoparticle exposure in AM using the fluorescent tag DHE (**E**). All three nanoparticle types produced significant amounts of ROS, but there was no difference between the nanoparticle types indicating that oxygen radicals could not account for any difference in AM toxicity seen with NB-2 exposure.

An alternative explanation for AM death, caused by the TiO_2 _NB-2 was also investigated. Reactive oxygen species (ROS) are often cited as the cause of many particle-induced cytotoxicities [20,21]. This possibility was examined two different ways. First, the peroxidation effect of TiO_2 _exposure on the membrane lipids was investigated using C_11_-BODIPY_(581-591) _loaded AM exposed to TiO_2 _nanoparticles. The resulting images are shown in Figure 5(a - d). All of the TiO_2 _nanoparticles tested caused some degree of lipid peroxidation indicated by green fluorescence (red fluorescence is the non-oxidized state).

Similarly, AM were loaded with dihydoethidium (DHE) to measure intracellular ROS directly and the results are presented in the Figure 5e. All of the TiO_2 _nanoparticles caused statistically significant increases in intracellular ROS compared to control AM over a 2-hour exposure period, but there was no significant difference between any of the individual nanoparticles for this effect indicating that the cytotoxicity caused by NB-2 could not be solely the direct result of ROS production. Other possible indicators of cell death that were investigated but not shown here were, nitrite release, peroxinitrite production, hydrogen peroxide production and superoxide anion release. All of these were negative for all TiO_2 _nanoparticles tested in AM culture (data not shown).

### The NB-2 Initiation of Inflammasomes

**Figure 6:**
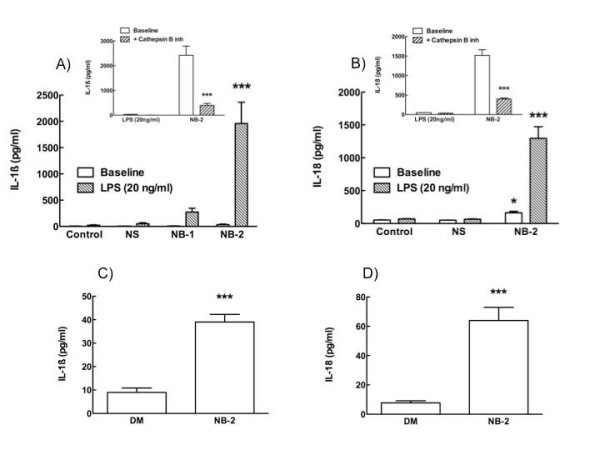
**Alveolar macrophages (AM) exposed to long TiO_2_nanobelts (NB-2) uniquely form the NALP3 inflammasome**. **A **and **B **panels show proxy measures for the NALP3 inflammasome formation, IL-1β and IL-18 are significantly enhanced by NB-2 exposure in the presence of a low concentration of the co-stimulant lipopolysaccaride (LPS). These increases were unique to NB-2 exposure in vitro, and they were significantly inhibited by 10 μM of the cathepsin B inhibitor CA-074 Me (respective **insets**). **B**, IL-18 was significantly increased in NB-2 exposed cells even with the absence of LPS co-stimulation. In panels **C **and **D**, these observations are supported by the *in vivo *observation of significantly increased IL-1β and IL-18 in the lavage fluid of mice 24 hours following NB-2 instillation (30 μg/mouse). Data expressed as mean ± *SEM*. Asterisk (*) indicates *P *< 0.05, double asterisks (**) indicates *P *< 0.01, and triple asterisks (***) indicates *P *< 0.001 compared to baseline or control production levels.

Inflammasomes are believed to be an early warning system for dangers to the innate immune system [22]. Figure 6 demonstrates how TiO_2 _NB-2 (long wire) exposure can uniquely affect AM cytokine production and cell signalling. Using a proxy measure for the NALP3 inflammasome, the NB-2 (100 μg/ml) significantly increased IL-1β and IL-18 production in the presence of sub-stimulatory amount (20 ng/ml) of LPS (Figures 6a and 6b). The LPS was necessary for the pro-forms of the cytokines to be present for caspase cleavage. No IL-33 release was detected by this treatment (data not shown). Increased IL-1β and IL-18 production were also measurable in vivo in lung lavage fluid 24 after instillation of NB-2 (Figures 6c and 6D). This inflammasome activation was significantly disrupted by the cathepsin B inhibitor peptide CA-074 Me as illustrated in the insets for Figures 6a and 6b, further implicating cathepsin B as an early initiator of TiO_2 _NB inflammation. The formation of the NALP3 inflammasome is consistent with other observations where AM are exposed to asbestos fibres or silica [23-25]. This may be a critical factor in the inflammatory and pathogenic properties of TiO_2 _NB-2 that are absent with the other forms of the TiO_2_. It is important to note that these nanoparticle exposures did not cause the same effects in virally transfected murine cell lines (RAW and MH-S tested, data not shown), and this was probably due to an inability to form the NALP3 inflammasome in these cells [26]. However, this effect is apparent in the human cell line THP-1 following phorbol ester (PMA) treatment and differentiation.

## Discussion

As seen in the list of toxicity studies presented in the background, there are a number of forms and/or shapes that the TiO_2 _particle can be manipulated by manufacturing techniques. The engineering aspect of TiO_2 _production increases these possibilities exponentially as the surfaces can be modified, the internal structures can be altered relative to the outside surface and the number of potential shapes becomes nearly limitless. The one shape of greatest concern to the toxicologist is the long wire or fibre. Fibres greater than 15 μm present a challenge to the macrophage, which is responsible for removing the foreign object from the body.

The AM, with its innate immune function, is responsible for binding, uptake and removal of inhaled material. A phenomenon referred to as "frustrated phagocytosis" describes an AM overcome by the unwieldy dimensions of a long fibre. This scenario usually applies to inhaled amphibole asbestos fibres, and the end result is that the fibres become biopersistent in the lung, because the fibres cannot be removed by the normal clearing processes.

The new generation of engineered nanofibres presents a justifiable concern to toxicologists. There is more than just a superficial resemblance of these manufactured wires to asbestos fibres. It would appear that the mechanism of how an AM deals with an asbestos fibre is identical or very similar to how an AM deals with a nanobelt or a nanowire made of TiO_2 _or other material for that matter. For example, Poland et.al., found that carbon nanotubes resembling asbestos produced asbestos-like pathology in mice [27]. This result was refuted in a more resent study on mesothelioma, which used a rat exposure model for MWCNT [28]. However, the end result (inflammation and disease) could be the same regardless of the material, depending more on the length of the material, rather than the composition. The dysfunction of AM particle processing results from an inability to sequester the fibre into a lysosome within the cell resulting in the subsequent release of cathepsin B and the formation of NALP3 inflammasome. This occurs with different forms of asbestos, silica, and it also occurs with TiO_2 _nanobelts longer than 15 μm.

Taken together, the data indicate that a relatively inert material such as TiO_2 _can become quite toxic and inflammatory when the material is designed to be longer than a lung macrophage can process. The term "frustrated phagocytosis" is simply a misnomer for a defective cell process, an inability to form functional lysosomes that leads to a cycle of cell death, inflammation, and eventually lung disease. The NB-2-induced inflammasome and the cathepsin B release presented in this study are also common to AM asbestos exposure and AM silica exposure. Every particle that caused these two events is also a particle that caused cell death in vitro, inflammation in vivo, and eventually some form of lung pathology such as fibrosis with long-term exposures.

## Conclusions

The engineers of these nanoparticles should always take into consideration the length of the particles they are creating. Eventually these particles could be the next occupational or environmental exposure of consequence.

## Methods

### Titanium Dioxide Synthesis

The nanobelts were synthesized as follows: 32 g NaOH was dissolved into 80 ml deionized water. Next, 1.2 g of anatase TiO_2 _particles was added to the 10 M NaOH aqueous solution. The mixture was vigorously stirred for 1 hour and then transferred to a 100 ml Teflon-lined stainless steel autoclave. The autoclave was sealed and put into a preheated oven to perform hydrothermal treatment at 200°C. After the hydrothermal processing, a white fluffy powder was obtained and washed with D. I. water and 0.1 M HCl. The washed samples were then calcinated at 700°C for 30 min at a ramp rate of 1°C/min for heating and cooling to get the long TiO_2 _nanobelts, while the short nanobelts were obtained at a heating ramp rate of 10°C/min due to the rupture caused by thermal-gradient-induced stress. For comparison tests, the TiO_2 _nanospheres were purchased directly from Alfa Cesar.

### Electron Microscopy (particles)

Scanning electron microscopy (SEM) images of the nanoparticles were done with a Hitachi S4700 field-emission scanning electron microscopy (SEM). The crystal structure of the TiO_2 _particles was characterized by X-ray diffraction with Cu *Kα *radiation (XRD, X'Pert Pro PW3040-Pro, Panalytical Inc.) and high resolution transmission electron microscopy (HRTEM), a 200 kV FEI/Philips CM20 apparatus). For tunnelling electron microscopy (TEM) sample preparation, the TiO_2 _powders were suspended in ethanol. The suspension was then dropped onto a holey carbon film supported by a copper grid, subsequently dried in air prior to TEM observation.

### Electron Microscopy (cells)

Macrophage suspensions were fixed in 2.5% EM grade glutaraldehyde in cacodylate buffer at pH 7.2. The cells were rinsed in dH_2_O and resuspended in 1% osmium tetroxide for 1 hr and rinsed in dH_2_O. For SEM imaging the cells were placed on a 0.6 um Millipore Isopore membrane filter followed by a graded ethanol series. Once in 100% ethanol the mounted cells were critically point dried in a Balzers CPD030, mounted on an aluminum stub, and sputter coated with gold/palladium in a Pelco Model 3 sputter coater. The cells were imaged in a Hitachi S4700 field emission scanning electron microscope at 10 kV. For TEM the cells were dried in a graded ethanol series followed by embedding of the cell pellet in epoxy. Thin sections were stained with 2% uranyl acetate for 30 min at room temperature, rinsed in dH_2_O, and stained for 5 minutes with Reynolds lead citrate stain. The cells were imaged in a Hitachi H-7100 transmission electron microscope at 75 kV.

### In vitro experiments

#### Animals

C57BL/6 (2-months old) were housed in controlled environmental conditions (22 ± 2°C; 30-40% humidity, 12-hour light: 12-hour dark cycle) and provided food and water *ad libitum*. All procedures were performed under protocols approved by the IACUC of the University of Montana.

#### Particles

Nanospheres and nanobelts were suspended in PBS/3.5% BSA solution. Nanospheres were sonicated for 1 minute and nanowires were sonicated briefly and vortexed for 1 minute.

#### Alveolar macrophage isolation

Mice were euthanized by sodium pentobarbital (Euthasol™), and the lungs with the heart were removed. Lung lavage was performed using ice-cold PBS (pH 7.4). Lung lavage cells were isolated by centrifugation (400 × g, 5 minutes, 4°C) and cell counts obtained using a Coulter Z1 particle counter (Beckman Coulter).

#### Cell culture

The cells were suspended in RPMI media supplemented with 10% fetal bovine serum, beta-mercapto ethanol, sodium pyruvate, supplemented with an antimycotic and antibiotics. Cells were suspended at 1 × 10^6 ^cells per ml and cultures were conducted in 96-well plates (24 hr adherent) or 1.5 ml microfuge tubes (4 hr suspension in Labquake shakers) in 37°C water-jacketed CO_2 _incubators (ThermoForma).

#### Toxicity and Assays

Cell viability was determined by trypan blue exclusion, and cell apoptosis was determined by Cell Death ELISA™ (Roche) according to the manufacturer's protocol. These assays used colorometric dyes, which were determined not to be affected by exposure to the titanium nanomaterials used in the experiments.

#### Bright Field Microscopy

Slides of alveolar macrophage cultures were prepared by centrifugation (1500 rpm, 5 min) in Shandon Cytospin II using 30 × 10^3 ^cells per slide and fixed/stained with HEMA 3 reagents obtained from ThermoFisher Scientific. Images were photographed with a Kodak digital camera attached to a Zeiss Axioskop at 600×.

#### Uptake Assay

The flow cytometry technique using side scatter to assess the amount of particle taken up by macrophage cells is described elsewhere [29].

#### Cytokine and Cathepsin Assays

Cytokine assays were performed according to the manufacturers' instructions (IL-1β, IL-18, and IL-33 R & D Systems). Cathepsin activity assay was performed on culture media (50 μl) mixed with 27 μM pan-cathepsin fluorogenic substrate (R & D Systems) for 1 hr at 37°C in a 96-well plate. The resulting fluorescence was captured by a Gemini plate reader (Molecular Devices) at 380 nm excitation and 460 nm emission. Fluorescent photomicrographs of lysosomes and cathepsin B were obtained in cells stained with acridine orange and cell-permeable cathepsin B fluorescent substrate respectively for 1 to 4 hours in culture.

#### ROS Assays

The lipid peroxidation imaging using C_11_-BODIPY_(581-591) _fluorescent stain were taken on a BioRad confocal microscope using the methods described elsewhere [30]. The assay for intracellular ROS involved culturing the isolated AM with nanoparticles in the Gemini plate reader warmed to 37°C. Dihydroethidium (DHE) was added prior to the start of the experiment and kinetic readings were taken throughout the 2-hour culture at 518 nm excitation and 605 nm emission wavelengths.

### In vivo experiments

Male C57BL/6J mice (2 months old) were obtained from Jackson Laboratories and were housed in controlled environmental conditions (22 ± 2°C; 30-40% humidity, 12-hour light: 12-hour dark cycle) and provided food and water *ad libitum*. All procedures were performed under protocols approved by the IACUC of CDC-NIOSH. The NIOSH animal program is accredited by the Association for Assessment and Accreditation of Laboratory Animal Care International. Nanospheres and nanobelts were suspended in dispersion medium (DM), which is PBS containing 0.6 mg/ml mouse serum albumin and 0.01 mg/ml 1,2-dipalmitoyl-sn-glycero-3-phosphocholine). Nanospheres were sonicated for 15 minutes (5 W output) and nanobelts were mechanically stirred for 3 hours prior to exposure. Mice were exposed to nanoparticles by pharyngeal aspiration. Mice were euthanized by sodium pentobarbital (Euthasol™), and a tracheal cannula was inserted. Lung lavages were performed using ice-cold PBS (pH 7.4) containing 5.5 mM D-glucose. Lung lavage fluid was isolated by centrifugation (650 × g, 5 minutes, 4°C) and stored at -20°C until the assays were conducted.

### Statistical Analyses

Statistical analyses involved comparison of means using a one or two-way ANOVA followed by Bonferroni's test to compensate for increased type I error. Statistical significance is a probability of type I error at less than 5% (P < 0.05). The minimum number of experimental replications was 3.

## Competing interests

The authors declare that they have no competing interests.

## Authors' contributions

RH and MB conducted the in vitro experiments. In addition, RH analyzed the data and prepared the manuscript and graphics. AH was responsible for the experimental direction of the in vitro experiments. DP and MW designed and performed in vivo experiments. NW conceived the experiments of nanobelt synthesis and characterization and provided the description of nanobelt synthesis and characterization. All authors have read and approved the final manuscript.
